# Copper Glufosinate-Based Metal–Organic Framework as a Novel Multifunctional Agrochemical

**DOI:** 10.1021/acsami.2c07113

**Published:** 2022-07-19

**Authors:** Beatriz Sierra-Serrano, Amalia García-García, Tania Hidalgo, Daniel Ruiz-Camino, Antonio Rodríguez-Diéguez, Georgiana Amariei, Roberto Rosal, Patricia Horcajada, Sara Rojas

**Affiliations:** † Department of Inorganic Chemistry, Faculty of Science, University of Granada, Av. Fuentenueva s/n, 18071 Granada, Spain; ‡ Advanced Porous Materials Unit, IMDEA Energy Institute, Av. Ramón de la Sagra 3, 28935 Móstoles, Madrid, Spain; § Department of Chemical Engineering, University of Alcalá, E-28871 Alcalá de Henares, Madrid, Spain

**Keywords:** metal−organic frameworks, agriculture, glufosinate, antibacterial activity, herbicide

## Abstract

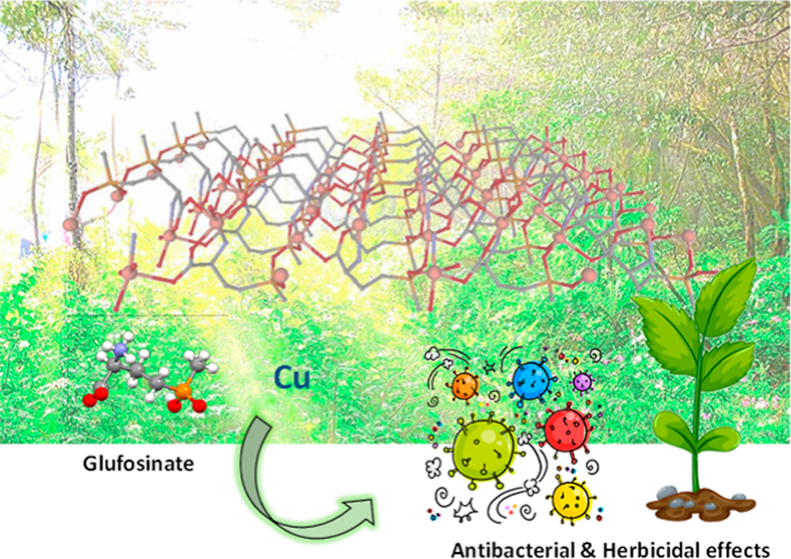

Pesticides are agrochemical compounds used to kill pests (insects, rodents, fungi, or unwanted plants), which are key to meet the world food demand. Regrettably, some important issues associated with their widespread/extensive use (contamination, bioaccumulation, and development of pest resistances) demand a reduction in the amount of pesticide applied in crop protection. Among the novel technologies used to combat the deterioration of our environment, metal–organic frameworks (MOFs) have emerged as innovative and promising materials in agroindustry since they possess several features (high porosity, functionalizable cavities, ecofriendly composition, *etc.*) that make them excellent candidates for the controlled release of pesticides. Moving toward a sustainable development, in this work, we originally describe the use of pesticides as building blocks for the MOF construction, leading to a new type of agricultural applied MOFs (or AgroMOFs). Particularly, we have prepared a novel 2D-MOF (namely, GR-MOF-7) based on the herbicide glufosinate and the widely used antibacterial and fungicide Cu^2+^. GR-MOF-7 crystallizes attaining a monoclinic *P*2_1_/*c* space group, and the asymmetric unit is composed of one independent Cu^2+^ ion and one molecule of the Glu^2–^ ligand. Considering the significant antibacterial activity of Cu-based compounds in agriculture, the potential combined bactericidal and herbicidal effect of GR-MOF-7 was investigated. GR-MOF-7 shows an important antibacterial activity against *Staphylococcus aureus* and *Escherichia coli* (involved in agricultural animal infections), improving the results obtained with its individual or even physical mixed precursors [glufosinate and Cu(NO_3_)_2_]. It is also an effective pesticide against germination and plant growth of the weed *Raphanus sativus*, an invasive species in berries and vines crops, demonstrating that the construction of MOFs based on herbicide and antibacterial/antifungal units is a promising strategy to achieve multifunctional agrochemicals. To the best of our knowledge, this first report on the synthesis of an MOF based on agrochemicals (what we have named AgroMOF) opens new ways on the safe and efficient MOF application in agriculture.

## 1 Introduction

Agrochemicals (mainly fertilizers and pesticides) have become a fundamental part of today’s agricultural systems to fulfil the huge demand of food. Although pesticide use is an old practice, the actual excessive usage of agrochemicals is deteriorating the quality of the ecosystems (living beings, soils, groundwaters), which strongly impacts on the public health and even leads to the development of new pesticide-resistant strains.^1−3^ Over the period 2011–18, the pesticides’ sales have risen [>350,000 tons per year only in the European Union (EU)], particularly, fungicides, bactericides, and herbicides, depending on the crop type. This important sale increase is closely associated with the land area occupied by agriculture, which is a limited resource (∼38% of the earth’s terrestrial surface; 1/3 – cropland, 2/3 – meadows and pastures for livestock).^4^ The major issues related with the extensive use of these agrochemicals, “*their ecological footprint*”, are as follows: (i) the projected population growth, which will require, by 2050, the overall agricultural production to increase by an astonishing 60%;^5^ (ii) the limited efficacy of the current pesticides on the market since a large proportion (10–75%) is not able to reach their target;^6,7^ (iii) their environmental toxic impact; (iv) the development of acquired resistances, with an estimated economic cost of 1.3 billion € only in the United States (US); and (v) their safety, which may result in both acute and chronic health problems.^8^

Although pesticides need to possess a toxicological effect to a specific target, the current challenge in agriculture is to sustain production and profitability using less toxic agrochemical inputs. Thus, the formulation development is a key initial phase in the ecological risk assessment of chemicals. In recent years, different nanocarriers have been proposed as vehicles for the controlled release of pesticides. Some are often considered “soft” or organic backbone nanoparticles (*e.g.*, polymers, lipids, nanoemulsions), but there are also examples of “hard” or rigid structure nanomaterials such as silica,^9−13^ clays,^14^ TiO_2_,^15^ carbon nanotubes,^16^ or graphene oxides.^17^ Recently, metal–organic frameworks (MOFs) have emerged as innovative and promising materials for the pesticide delivery.^100^ MOFs are regarded as a unique class of porous coordination polymers, comprising inorganic nodes (*e.g.*, atoms, clusters, chains) and organic polydentate linkers (*e.g.*, carboxylates, azolates) that assemble into multidimensional porous periodic lattices.^18^ In particular, certain MOFs have proven several features that make them excellent candidates for environmental applications such as (i) large pore surfaces and volumes associated with high sorption capacities;^19^ (ii) active sites, where adsorbates can be anchored; (iii) easily functionalizable cavities, where specific host–guest interactions may take place; (iv) adsorbate release controlled by adsorbate–matrix interactions, diffusion, and/or matrix degradation; (v) the possibility of using biofriendly or active constituents (cations or organic linkers) as part of their structure and non-toxic solvents during their synthesis; (vi) *a priori* environmentally friendly character; and (vii) (bio)degradability, remaining stable to carry out their functions, and then, being eliminated, preventing accumulation and potential animals/plants side effects.^20^ Particularly, MOFs have been recently investigated for the controlled delivery of pesticides. For instance, a Ca-l-lactate material was proposed for the release of the fumigant *cis*-1,3-dichloropropene,^21^ the iron(III) trimesate MIL-100(Fe) for the release of the fungicide azoxystrobin,^22^ and the composite chromium(III) terephatalate MIL-101@CMCS (CMCS: carboxymethyl chitosan) as the delivery agent of the insecticide dinotefuran.^23^

In this work, we want to go a step further in the promising role of MOFs in agriculture: using for the first time pesticides as building blocks for the MOF construction. Pesticide-based MOFs would be attractive formulation prototypes for the targeted multiple release of pesticides since (i) active ingredients (AIs) are a constitutive part of the matrix itself, insuring high loadings of the AIs and avoiding multistep procedures to load them (“*one-pot synthesis*”); (ii) both the cation and organic linker can be “*active*”, mimicking bioagrochemicals (based on many active ingredients) achieving a *combined/synergic effect* and reducing the potential development of resistances; and (iii) they are “*economically appealing*” since one formulation will have multiple actions and they will be based on *low cost* and *eco-friendly* reactants. In this sense, we have designed here a novel multifunctional MOF with both herbicide and antibacterial capacities. For this aim, copper, one of the eight essential plant micronutrients, was selected as the constitutive cation. About 70% of Cu in plants is found in chlorophyll; its deficiency in crop plants results in early aging or lowered levels of chlorophyll, which leads to yield reduction that goes unnoticed if the deficiency is not severe.^24^ Cu^2+^ is not only used as a fertilizer in agriculture but also in the management of plant diseases due to its recognized antifouling, antifungal, and antibacterial properties.^25^ On the other hand, we have selected the naturally occurring herbicide glufosinate (or phosphinothricin) as a ligand. Glufosinate targets glutamine synthetase, the second most abundant enzyme in plant leaves, essential for nitrogen metabolism by catalyzing the adenosine triphosphate-dependent incorporation of ammonia into glutamate to yield glutamine.^26,27^ Glufosinate is a contact herbicide with limited translocation, making it effective primarily on annual weed species. It also tends to provide lower activity on larger weeds compared to seedlings, and it is recommended to spray on small weeds.^28^ Further, glufosinate is a key herbicide to manage glyphosate-resistant weeds as it is a broad-spectrum herbicide. High applications rates (1470 g·ha^–1^·year^–1^) are needed as glufosinate persistence in the environment is the shortest compared to other herbicides.^29^ Glufosinate has never been loaded in a nanocarrier for its controlled release. Particularly, as a reactant for the MOF preparation, glufosinate is a flexible phosphinic acid showing three potential metal-binding sites (*i.e.*, phosphinic, amino, and carboxylic groups), which will *a priori* facilitate the formation of MOF structures. Moreover, glufosinate is structurally similar to 2-methylglutarate, a ligand previously used by some of us in the synthesis of a large family of flexible copper-based MOFs (GR-MOF-3 and GR-MOF-5).^30,31^ The plasticity of the metal atoms and the fact that there are many atoms with sp^3^ hybridization offer the possibility to build multidimensional structures with different topologies.

Thus, this article reports the synthesis and characterization of a novel 2D-MOF (namely, GR-MOF-7) based on the herbicide glufosinate and the widely used antibacterial and fungicide Cu^2+^, evaluating its multiple action against different pesticides. Achieving this kind of multitargetting material, never explored up to date, might open a new field in the preparation of more efficient pesticides as fascinating multifunctional materials.

## 2 Results and Discussion

### 2.1 Synthesis and Crystal Structure Description of GR-MOF-7

**Figure 1:**
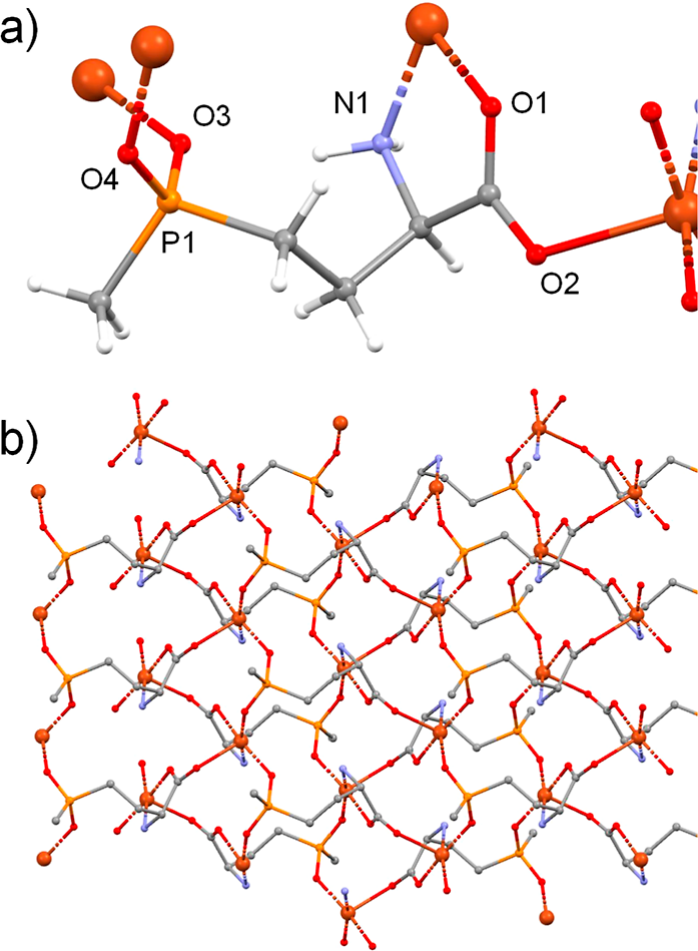
Crystal structure of GR-MOF-7. (a) Coordination mode of the glufosinate ligand that coordinates to four Cu^2+^ ions due to all its donor atoms. (b) View of the sheet along the *a* crystallographic axis. Hydrogen atoms have been omitted for clarity.

For the first time, a glufosinate-based MOF (named GR-MOF-7) based on the pesticide glufosinate and the antibacterial/antifungal copper was successfully isolated upon exhaustive optimization of the solvothermal reaction conditions. Briefly, a reactive aqueous/ethanolic mixture composed of Cu(NO_3_)_2_·3H_2_O and glufosinate ammonium (HNH_4_Glu) in a 1:1 molar ratio was heated at 100 °C for 24 h (see the Supporting Information, Section S1), leading to the formation of the 2D GR-MOF-7 structure (Tables S1–S3 and Figures 1 and S2 in the Supporting Information). GR-MOF-7, formulated as [CuC_5_H_10_NO_4_P], was prepared in high purity as large single crystals [∼1 μm; see scan microscopy images (SEM) in Figure S3 in the Supporting Information] suitable for its structure resolution by single-crystal X-ray diffraction (SCXRD). As revealed by SCXRD, GR-MOF-7 crystallizes attaining a monoclinic *P*2_1_/*c* space group and the following unit cell parameters: *a* = 9.985(3), *b* = 4.9674(15), *c* = 16.103(4) Å, α = γ = 90, and β = 106.876(10)°. The asymmetric unit is composed of one independent Cu^2+^ ion and one molecule of Glu^2–^ ligand. Each metal center shows a distorted square pyramid geometry, in which the planar positions are occupied by a nitrogen atom from the amino group, two oxygens from the phosphinic group, and a third oxygen belonging to a carboxylic group. The apical position is occupied by one oxygen from a carboxylic group pertaining to a different glufosinate ligand (Figure 1a). Each Cu^2+^ ion is coordinated to four glufosinate ligands as a whole. The Cu–N bond distance is 1.977(4) Å, while the Cu–O bond lengths range from 1.935(3) Å for oxygens in planar positions to 2.274(3) Å for O_2_ atoms in the apical position due to Jahn–Teller distortion. The ligand coordinates for all its donor atoms by both monodentate and bidentate modes. This disposition of the ligands creates a two-dimensional structure in which sheets grow parallel to the *bc* plane (Figure 1b). The layers do not interact between themselves, but intramolecular hydrogen bonds exist between the primary amine and the oxygen from the phosphinic group of the same glufosinate ligand and the oxygen belonging to a carboxylate group of a different ligand molecule with distances of 2.941 and 2.932 Å, respectively (Table S3 in the Supporting Information).

**Figure 2:**
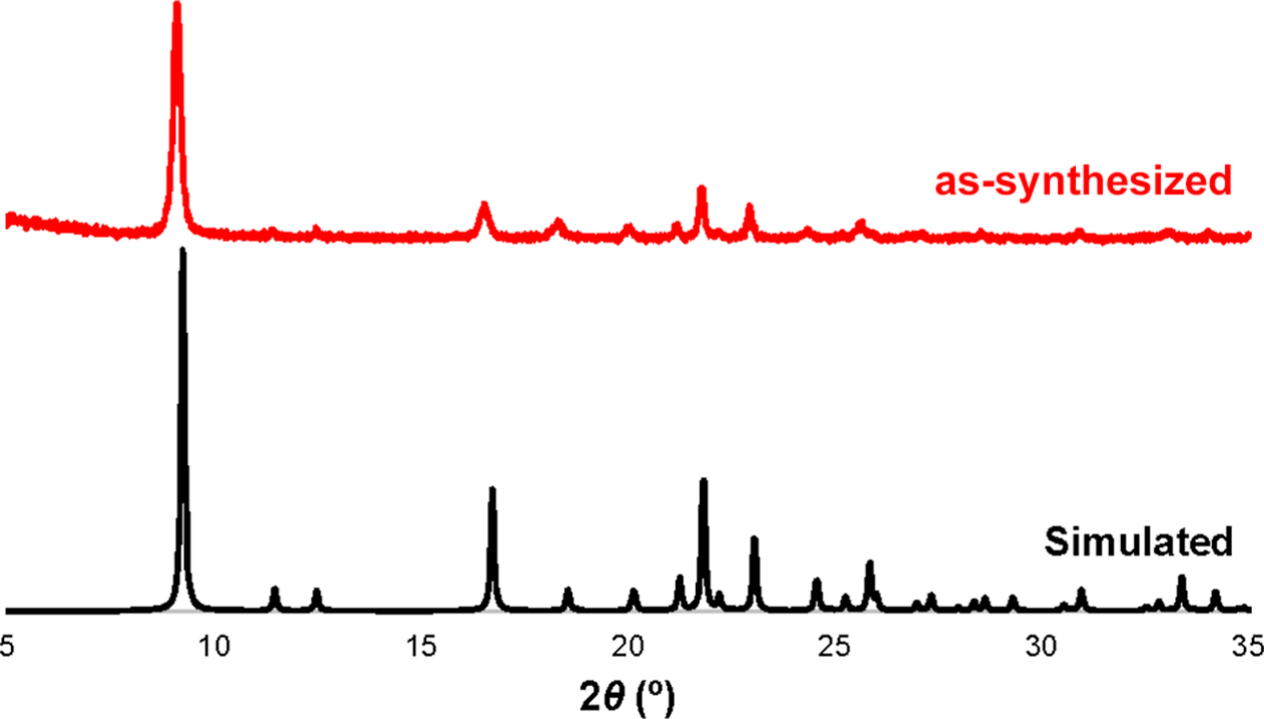
Powder and single-crystal XRD patterns of the GR-MOF-7 material.

GR-MOF-7 was successfully scaled up 10 times, both avoiding the pressure (solvothermal *vs* reflux conditions) and reducing the synthesis time (24 *vs* 2 h), leading to a high yield (∼60%) powdered material at the gram scale (*ca.* 0.2 g). The characteristic crystalline phase of GR-MOF-7 was identified in the scaled-up bulk sample by comparing both the location and intensity of the main Bragg reflections with those of the crystalline structure resolved by SCXRD (Figure 2). To check the phase purity, the Le Bail fitting was carried out using the unit cell parameters of the GR-MOF-7 structure (Figure S4 in the Supporting Information). As observed in Figure S4, not even a trace of any other impurity phase is present in the pristine sample.

### 2.2 Physicochemical Characterization and Stability Studies

A significant challenge on the use of MOFs in agriculture is to fully characterize them, including the evaluation of their chemical and structural stability (usually studied by XRD) under working conditions. Regarding the characterization of GR-MOF-7, when compared with the free linker, the Fourier-transformed infrared spectrum of GR-MOF-7 shows a better definition of the bands at *ca.* 3145 and 3240 cm^–1^ corresponding to ν_NH_ coming from the −NH_2_ groups, whose rotation might be more restricted than in the free state (free linker, Figure S5). Further, characteristic peaks of the phosphinate group (normally found at 1033 and 1115 cm^–1^), representing the O–P–O symmetric and asymmetric stretching vibrations (ν_PO2s_ & ν_PO2_), are also better defined and slightly shifted to 1049 and 1131 cm^–1^ in comparison with the free ligand, respectively.^32^ The peak at 1637 cm^–1^, assigned to the carboxylic acid C=O stretching (ν_C=O_), and the two bands at 1527 and 1596 cm^–1^, associated, respectively, with asymmetric and symmetric stretching of the COO group (ν_COOas_ and ν_COOs_), are also shifted to 1606 and 1635 cm^–1^. This fact suggests the coordination of Cu to the −P(CH_3_)O_2_H, −NH_2_, and −COOH groups by the deprotonation of some acid groups, which is in agreement with the structural data (see above).

The chemical composition of GR-MOF-7, [CuC_5_H_10_NO_4_P], was also confirmed by elemental analysis (C, H, and N), showing Theo. (%): C, 5.77; H, 4.15; N, 5.77, which fits well with the Exp. (%): C, 6.03; H, 4.55; N, 6.03. Further, the chemical composition was further compared with the thermal degradation product. Thermogravimetric analysis shows a first slight weight loss (from room temperature to 100 °C; 0.3 wt %, Figure S6), attributed to the water adsorbed on the external surface of the crystals. The following progressive mass loss, starting at 250 °C, can be assigned to the GR-MOF-7 decomposition associated with the organic ligand oxidation. The final residue was identified as a mixture of Cu_2_O_7_P_2_ and Cu_3_O_7_P_2_ in a ratio close to 85:15 (DIFFRAC.EVA, Bruker, Figure S7).

On the other hand, pesticides are usually sprayed as an aqueous solution or suspension in the fields in order to reach different parts of plants or the ground. In this sense, a complete physicochemical characterization (colloidal, chemical, and structural stability) of GR-MOF-7 was performed in the presence of water. First, the colloidal stability of the as-prepared GR-MOF-7 was confirmed by suspending the material in an aqueous solution for 24 h (Figure S8). After an initial slight aggregation (from 66 ± 10 to 252 ± 45 nm in 2 h), GR-MOF-7 remains at the nanometric range with a constant negative charge (average size: ∼223 ± 43 nm; ζ-potential: −9 ± 4 mV) for 24 h, confirming the stability of this colloidal solution. Further, the aqueous chemical stability of the prepared compound was investigated by UV–vis spectroscopy and proton nuclear magnetic resonance spectroscopy (^1^H NMR) in an attempt to determine the potential linker leaching. After 24 h of contact time, no signal was detected in the recorded ^1^H NMR spectra of GR-MOF-7 (paramagnetic compound), observing sharp and defined peaks of the free glufosinate (Figure S9). This behavior was also confirmed by UV–vis, demonstrating the stability of the Cu–glufosinate bond in this medium, not only at 24 h but also up to 5 days in aqueous solution (Figure S10). Finally, the structural stability of the GR-MOF-7 aqueous suspension was confirmed by powder XRD (PXRD) (Figure S11). In this case, GR-MOF-7 was stable up to 24 h, despite the emerging of new diffraction peaks after 11 h. This effect could be related to the formation of new phases since the integrity of the whole frame has not been affected.

### 2.3 Antibacterial Effect of GR-MOF-7

Considering the substantial antibacterial activity of Cu-based compounds in agriculture (revolutionized crop protection in the 20^th^ century),^25^ the potential combined bactericidal and herbicidal effect of GR-MOF-7 was investigated, evaluating first their biological activity against most common foodborne bacteria. In this sense, *Staphylococcus aureus* (SA) and *Escherichia coli* (EC) were selected as main representatives of Gram-positive and Gram-negative bacterial strains, respectively. Both are bacterial pathogens responsible for infections in humans and various species of wild, companion, and agricultural animals.^33^ The zoonosis capacity of these microorganisms (estimated in ∼60% transmitted pathogens between humans and livestock) is due to the specific bacterium features (*i.e.*, photoadaptative clonal lineages) as well as modern agricultural practices (*e.g.*, globalization, ubiquitous use of antibiotics).^34^ The GR-MOF-7 antibacterial activity was determined by the bacteria viability, following both the colony-forming units (CFU) and the microbial enzymatic activity using the fluorescein diacetate hydrolysis assay (FDA, see Supporting Information Section S1 for further details). A large range of GR-MOF-7 concentrations (from 0 to 250 ppm) were initially tested in order to select the most effective dose. Remarkably, a high and concentration-dependent antibacterial effect was evidenced for both strains after 20 h incubation, with a growth inhibition of *ca.* 40 and 24% of SA and EC, respectively, when using very low concentrations of GR-MOF-7 (≤2.5 ppm; Figure S12A). The minimum inhibitory concentration (MIC), or the lowest concentration able to prevent growth of a bacterium, was then estimated to be 1 and 2.5 ppm of GR-MOF-7 for the SA and EC, respectively, corresponding to a decrease of the CFU values to almost 0 (Figure S12B). In fact, these low values could be beneficial since the maximum permitted rates by the EU regulations are limited at ∼12 and 3 mg·kg^–1^ (ppm) for Cu^2+^ and glufosinate commercial agents, respectively.^25,27,35^

**Figure 3:**
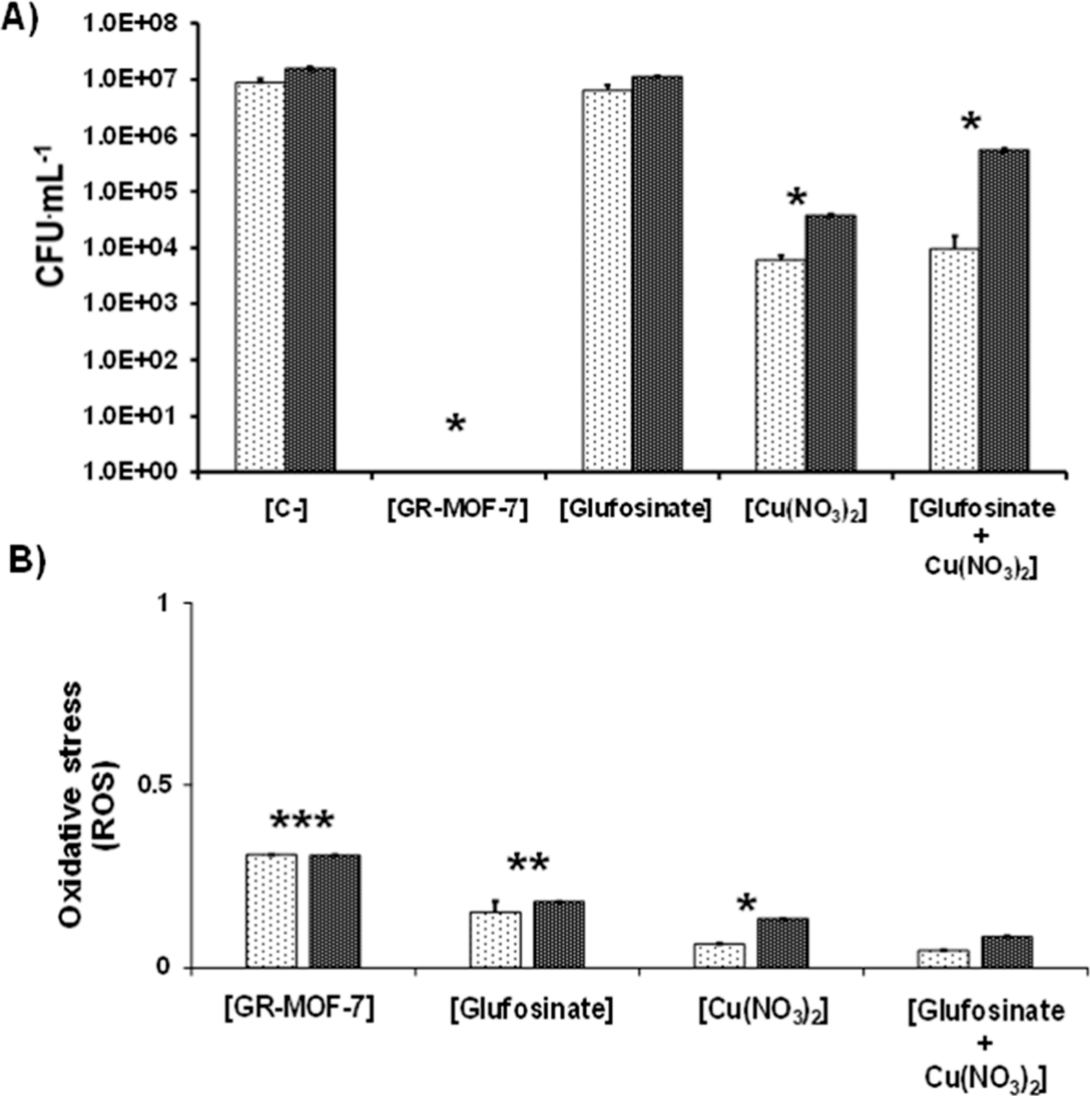
Colony-forming unit (A) and ROS induction (B) of SA (white column) and EC (black column) after 20 h of contact with the selected active GR-MOF-7 concentration together with the corresponding amount of the following controls: Cu(NO_3_)_2_, free glufosinate, and a precursor mixture. In all cases, each sample value was normalized with a negative control (C–, 100% of bacterial viability). The statistical significance was disclosed as *p < 0.05; **p < 0.01; ***p < 0.005.

Once a low MIC of GR-MOF-7 was evidenced for each strain, the subsequent step was to shed light on the main action mechanism triggered along with the role of each constituent in this procedure. One of the most renowned growth inhibition processes is the generation of reactive oxygen species (known as ROS), associated to the formation of HO^•^, O_2_^•–^, and HO_2_^•^ species, among others, which results in bacterial death.^36,37^ To shed some light on the bactericidal mechanism, the antibacterial profile (CFU, FDA) as well as the ROS production of the most active bacterial dose was investigated in comparison with its precursors using the same proportion as the bulk material [individually or in a physical mixture format: Cu(NO_3_)_2_, glufosinate and glufosinate + Cu(NO_3_)_2_; Figures 3 and S13]. In agreement with previous viability assays, GR-MOF-7 maintained its high antibacterial effect in both strains [lack of any CFU formation—statistically significant (*p* < 0.01) with 39 and 25% of SA and EC enzymatic inhibition, respectively]. Note here that while free glufosinate does not significantly affect the CFU value of both bacteria, only a slight repercussion is observed in the presence of the isolated Cu^2+^ constituent (CFU decrease, 13% SA–29% EC of inhibition). Even further, GR-MOF-7 exhibits a much higher antibacterial activity than the physical mixture of glufosinate + Cu(NO_3_)_2_ (CFU decrease, 0% SA–36% EC of inhibition), evidencing that not only the component nature but also the relevance of the glufosinate and copper association/interactions within the material is important.

**Figure 4:**
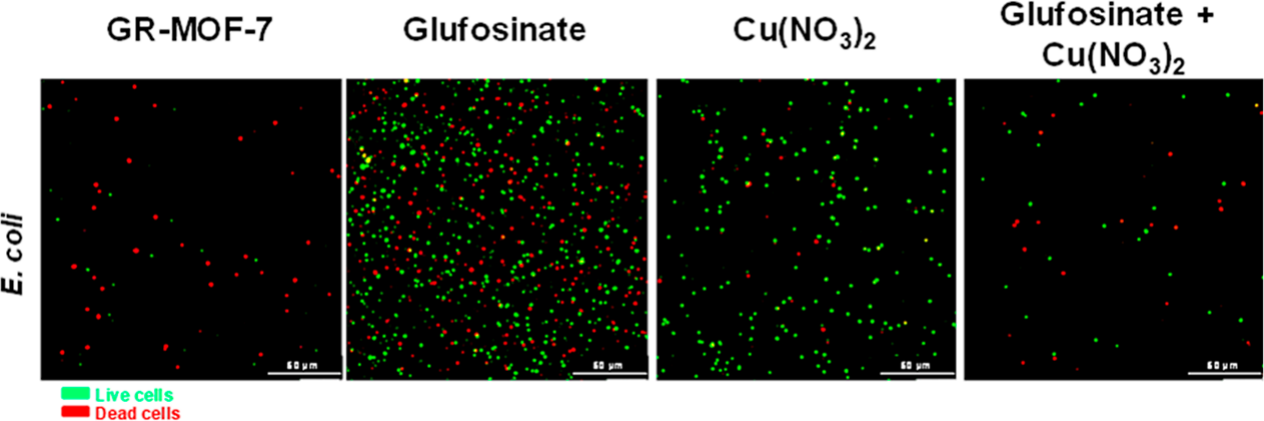
Fluorescence LIVE/DEAD confocal images of sessile SA and EC on the cover glass surface after 20 h of contact with the GR-MOF-7 compound and its constituents; Cu(NO_3_)_2_, free glufosinate, and a precursor mixture are used as controls. The scale bar corresponds to 50 μm. All the images were taken at 63×.

For a better understanding of this biocidal effect, the live and dead bacteria (“*green*” and “*red*” labels, respectively) were discriminated by a LIVE/DEAD staining (and observed with confocal microscopy), evaluating simultaneously the potential ROS induction in the presence of diverse treatments. Remarkably, upon 20 h incubation, we evidenced the absence of viability in the few remaining bacteria, decreasing not only the number of live cells but also the dead ones, which suggest a stronger effect (Figures 4 and S14). In the case of the precursors, the linker itself did not show any toxicity, observing a slight reduction of the bacteria presence after the incubation with the Cu salt (individually and in a mixture format).

Concerning the ROS production, similar levels of ROS were observed for both bacteria after 20 h of incubation, requiring a lower dose in the case of SA (∼0.3 normalized value for 1 and 2.5 ppm in SA *vs* EC in comparison with the negative control C–, respectively). The pattern exhibited by its constituents also reflects an influence on the bacterial oxidation balance, leading to a dual ROS production for both the ligand and the metal, regardless of the interaction format (individual species or in a mixture). In all cases, the ROS values showed statistically significant differences in comparison with the negative control (normalized here as no ROS production with a value = 1), being higher in the case of GR-MOF-7 with *p* < 0.005 and even with its own precursors (*p* < 0.01). This observation suggests that the higher antibacterial effect of GR-MOF-7 could be associated with the generation of an important ROS production. On the other hand, the largest effect displayed in SA strain could be due to its most soft structure (absence of capsule formation) compared to EC (typically with a rigid cellular wall), which might also hamper the penetration and retention of chemicals.^38^

### 2.4 Evaluation of the Herbicide Activity of GR-MOF-7

In order to test its potential as a multitarget material (bacteria and pest), the effectiveness of GR-MOF-7 as a herbicide was tested against the model weed species *Raphanus sativus* (radish) as it is considered an invasive species in berries and vines crops and glufosinate is normally used against this weed.^39^ Through this assay, the herbicide impact on diverse development stages of a plant was evaluated by means of seed germination and plant growth. Concerning the effect of GR-MOF-7 on seed germination, the active concentration of glufosinate (0.01 M) was first determined following the recommendations of the commercial glufosinate pesticide BASF-Rely280 (for further details, see the Supporting Information, Section S1).^39^ When using this concentration, GR-MOF-7 fully inhibited the seed germination (100 ± 0% of seed germination reduction), showing a greater herbicide effect than the free glufosinate (32 ± 7% of seed germination reduction), demonstrating the combined herbicide effect of glufosinate and Cu in the inhibition of radish seed germination. However, it should be noted that the 100% reduction of germination inhibition was also reached when using the physical mixture of glufosinate + Cu(NO_3_)_2_. In contrast with previous antibacterial observations, here, the nature of the components might have a more relevant effect than their interactions.

**Figure 5:**
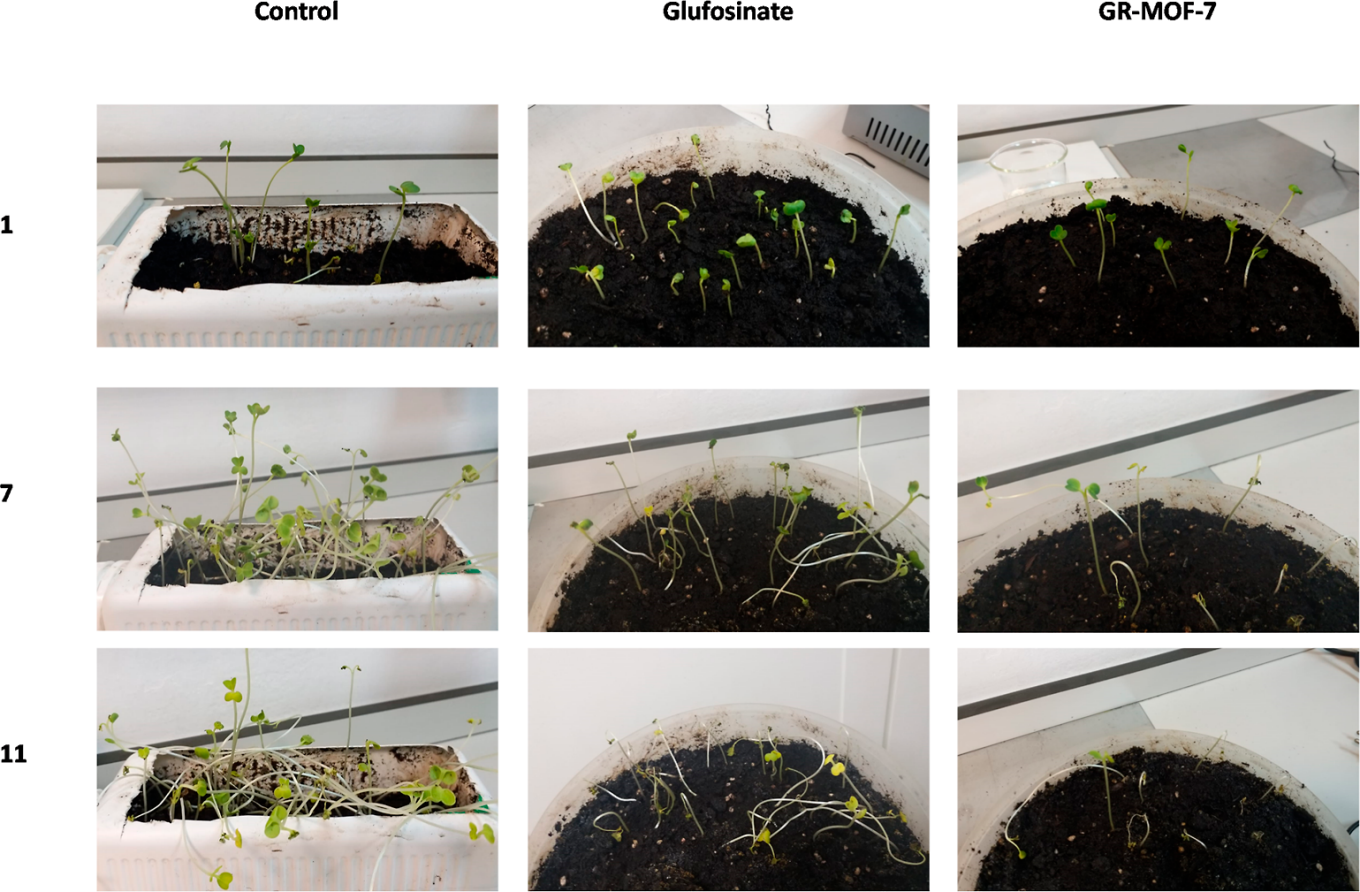
Effect of free glufosinate and GR-MOF-7 on grown radish plants. Detailed day-by-day pictures of the treated plants are shown in the Supporting Information.

Finally, the effect of GR-MOF-7 in plant growth was studied in order to check the applicability of this compound in the field. When the three-leaf stage was reached, *R. sativus* plants were sprayed once with a GR-MOF-7 active solution (1 mL, 0.01 M; *t* = 0). Radish plants treated with GR-MOF-7 were fully dry after 8 ± 1 days (Figure 5). On the other hand, there is no significant difference between the control and free glufosinate groups during 7 days of study, which reflects the absence of plant toxicity at the selected dose (0.01 M). These results demonstrated that a lower concentration is needed to inhibit plant growth inhibition when compared GR-MOF-7 with free glufosinate.

After 7 days of MOF treatment, leaves started to turn yellow and stems became fragile until day 11, when plants were fully dry. These results clearly indicate the greater herbicide effect of GR-MOF-7 compared to free glufosinate, where the former shows more effectiveness of drying radish plants with the same concentration of the active ingredient. Finally, in an attempt to assess the safety use of this novel material in crops, GR-MOF-7 was tested against a non-targeted plant (*Ribes nigrum*, berry, Figure S16). No signs of toxicity, evaluated as the drying effect, were observed for 11 days. This observation demonstrates the selectivity of GR-MOF-7, which could be used to control bacteria and weed plants without damaging crops.

## 3 Conclusions

A novel pesticide/antifungal/antibacterial-based MOF is here presented for the first time as an attractive formulation prototype for the targeted multiple release of agrochemicals. The copper(II) glufosinate GR-MOF-7 was easily and efficiently (0.2 g in 2 h) synthetized by a simple green method using accessible reactants. The solid, exhibiting Cu^2+^ with a square pyramid geometry, a nitrogen from the amino group, two oxygens from the phosphinic group, and a third oxygen belonging to a carboxylic group, is quite soluble in water (2.55 g·L^–1^) and shows a good water stability (up to 5 days).

Interestingly, GR-MOF-7 exhibits an important antibacterial activity against SA and EC (involved in agricultural animal infections), improving the results obtained with its individual precursors or even physical mixed precursors [glufosinate and Cu(NO_3_)_2_]. On the other hand, this material is an effective pesticide against the germination and the plant growth of the weed *R. sativus*, an invasive species in berries and vines crops, demonstrating that the construction of MOFs based on herbicide and antibacterial/antifungal units is a promising strategy to achieve multifunctional agrochemicals. On the whole, in contrast with the isolated constituents (glufosinate and copper), the GR-MOF-7 material exhibits a biocompatible character toward plant crops with a selective 3 in 1 effect, combining enhanced antibacterial, herbicidal, and fertilizing properties.

## Supporting Information Available

Crystallographic data of GR-MOF-7, spectral data, additional structural figures, materials and methods, physicochemical characterization, and antibacterial results (PDF)
