# Supplementary material for: Copper Glufosinate-Based Metal–Organic Framework as a Novel Multifunctional Agrochemical
Source: ACS Appl Mater Interfaces. 2022 Jul 19;14(30):34955–62. doi: 10.1021/acsami.2c07113 (PMC9354010; doi:10.1021/acsami.2c07113)
Supplement: Supplementary file 1 — am2c07113_si_001.pdf [file am2c07113_si_001.pdf]

A copper glufosinate based Metal-Organic Framework as novel multifunctional agrochemical

Supporting information

Beatriz Sierra-Serrano,[a] Amalia García-García,[a] Tania Hidalgo,[b] Daniel Ruiz-Camino,[b] Antonio Rodríguez-Diéguez,[a] Georgiana Amariei,[c] Roberto Rosal,[c] Patricia Horcajada,[b,*] Sara Rojas[a,*]

[a] Department of Inorganic Chemistry, Faculty of Science, University of Granada. Av. Fuentenueva s/n, 18071 Granada, Spain

[b] Advanced Porous Materials Unit, IMDEA Energy Institute. Av. Ramón de la Sagra 3, 28935 Móstoles-Madrid, Spain

[c] Department of Chemical Engineering, University of Alcalá, E-28871 Alcalá de Henares, Madrid, Spain

*srojas@ugr.es, patricia.horcajada@imdea.org

Table of content

S1. Materials and Methods……………………………………………………………………..S2-S5

S2. Crystallographic data………………………………………………………………………S6-S8

S3. GR-MOF-7 characterization………………………………………………………………S9-S11

S4. Aqueous stability studies of GR-MOF-7……………………………………………..S12-S14

S5. Antibacterial effect……………………………………………………………………………S15-S16

S7. Herbicidal effect………………………………………………………………………………..S17

S6. References……………………………………………………………………………………….S19

S1. Materials and Methods

Reagents and solvents

Chemicals were readily available from commercial sources and used as received without further purification.

Synthesis of GR-MOF-7 ([$CuC_5H_{10}NO_4P$])

10 mg (0.051 mmol) of glufosinate ammonium (or 2-amino-4-(hydroxymethylphosphinyl)butyric acid ammonium salt) were dissolved in 0.5 mL of distilled water. Then, 1.5 mL of ethanol was added to the glufosinate solution. In a separate vial, 13 mg (0.054 mmol) of $Cu(NO_3)_2{\cdot}3H_2O$ were dissolved in 0.5 mL of distilled water. Similarly, once the metal salt was dissolved, 1.5 mL of ethanol metal solution were added dropwise to the ligand solution. The resulting bluish mixture was placed in a closed glass vessel and heated in an oven at 100 ºC for 24 h. X-ray quality crystals of GR-MOF-7 were obtained during the heating process under autogenous pressure and washed with water. Yield: 49.79%. Anal Calcd. for [$CuC_5H_{10}NO_4P$]: C, 5.77; H, 4.15; N, 5.77. Found: C, 6.03; H, 4.55; N, 6.03.

Scale up of GR-MOF-7

0.100 g (0.505 mmol) of glufosinate ammonium and 0.106 g (0.440 mmol) of $Cu(NO_3)_2{\cdot}3H_2O$ were refluxed in a mixture of 10 mL of distilled water and 30 mL of ethanol for 2 h. After cooling down to room temperature (RT), the suspension was filtered and washed with distilled water. Yield: 59 ± 5%.

General instrumentation

Elemental analyses were carried out on a Thermo Scientific analyser model Flash 2000. Fourier transformed infrared (FTIR) spectra were measured in the 4000 to 400 cm^{-1} range using a Bruker Tensor 27 FT-IR with an ATR accessory instrument and Opus data collection program. PXRD diffractograms were registered in a Bruker D8 Advanced equipment. Thermogravimetric analyses (TGA) were carried out using a Shimadzu mod. DXC-50Q, at a heating rate of 20 ºC·min^{-1}. UV-vis spectra were collected on a Thermo Unicam UV 300. ^1H NMR were acquired on a 500 MHz Varian Equipment using deuterated water as solvent. Scanning electron microscopy (SEM) was carried out using a Hitachi S510 microscopy at 25 kV coupled with a SE detector of 7 nm at 25 kV. For the antibacterial activity, we have measured the optical density (OD) using a Shimadzu UV-1800 spectrophotometer, CFU estimations were counted using a CL-1110 counting instrument (Acequilabs, Spain); for the determination of the enzymatic activity loss, a Fluoroskan Ascent FL Fluorimeter/Luminometer (Thermo Scientific, Waltham, MA, USA) was used, whereas the visualization of this activity was performed by using a Leica Microsystems Confocal SP5 Fluorescence microscope (Germany).

Single-crystal structure determination

Single crystals of suitable dimensions were used for data collection. For GR-MOF-7, diffraction intensities were recorded on a Bruker X8 APEX II and Bruker D8 Venture with a Photon detector (Bruker, Madrid, Spain) equipped with graphite monochromated MoKα radiation (λ = 0.71073 Å). The data reduction was performed with the APEX2 software[1] and corrected for absorption using SADABS.[2] The structure was solved by direct methods and refined by full-matrix least-squares with SHELXL-2018.[3] The main refinement parameters are listed in **Table S1** in the

Supporting information (SI). Details of selected bond lengths and angles are given in **Table S2**. CCDC reference number for the structure is 2109530.

Water stability studies

UV-vis spectra were recorded to investigate the stability of GR-MOF-7 in water at pH = 6. Solutions were prepared via dilution of a 50 μM GR-MOF-7 stock. Further, the stability of GR-MOF-7 was confirmed by ^{1}H NMR, by dissolving a small amount of GR-MOF-7 and glufosinate linker (*ca.* 5 mg) in deuterated water for 24 h. The particle size and ζ-potential determinations were performed using a Malvern Nano-ZS, Zetasizer Nano series. Aroun 1 mg of GR-MOF-7 was dispersed in 1 mL of water using an ultrasound tip (10% amplitude, 30 s) for 1 min. Size and ζ-potential were analyzed in triplicate (n = 3).

6 mg of GR-MOF-7 was loaded in a 1.27 mm polyimida capillary (95820-11; Cole-Parmer, Illinois, USA), humidified with 50 µL of distilled water, and mounted on a spinner rotating at about 5 Hz (V= 45 kV, I= 40 mA) to improve the particles' statistics. Diffraction data were collected each hour over a 24 h time period in a Bruker D8 Advanced equipment.

Antibacterial activity

The Gram-positive *Staphylococcus aureus* (CECT 240, strain designation ATCC 6538P) and Gram-negative *Escherichia coli* (CECT 516, strain designation ATCC 8739) bacteria were used as reference strains for the antibacterial activity tests. The microorganisms were preserved at −80ºC in glycerol (20% v/v) until their use. Reactivation was performed in nutrient broth (1 mL of inoculums in 20 mL of NB; composed by 5 $g{\cdot}L^{-1}$ beef extract, 10 $g{\cdot}L^{-1}$ peptone, 5 $g{\cdot}L^{-1}$ NaCl, pH=7-7.2) at 37 °C under stirring (100 rpm) and routinely tracked by measuring OD at 600 nm (Shimadzu UV-1800 spectrophotometer) to preserve the exponentially growing phase of the microorganisms during the total time of contact (20 h). Innocuous of 10^{6} $cells{\cdot}mL^{-1}$ of both bacteria were prepared in 1/500 NB.
For the antibacterial experiments, diverse concentrations of GR-MOF-7 solutions (0, 0.5, 1, 1.5, 2.5, 3.5, 4.5, 5, 25, 50, 100, 250 ppm) were prepared as well as control samples (glufosinate, $Cu(NO_3)_3{\cdot}3H_2O$, and glufosinate + $Cu(NO_3)_3{\cdot}3H_2O$), being dissolved over 2.4 mL of the previously mentioned 10^{6} $cells{\cdot}mL^{-1}$ inoculums of a 24-well plate. In the case of the controls, for each desired concentration, the quantity of each constituent was adjusted to the corresponding part of the bulk MOF (*e.g.,* 1 ppm of GR-MOF-7 corresponds to 0.65 ppm of glufosinate + 1 ppm of $Cu(NO_3)_3{\cdot}3H_2O$). After 20 h of incubation at 37ºC without stirring, the *bacterial viability* was evaluated by determining:

i) colony-forming units (CFU), where bacteria aliquots were placed in sterile 96 well plates in 10-fold serial dilutions in phosphate-buffered solution (PBS). Replicated 10 µL spots were placed on Petri dishes containing NB agar-medium and after 24 h incubation at 37 °C without stirring, CFU were counted using a CL-1110 counting instrument (Acequilabs, Spain). For colony number estimations, at least three replicates of at least two serial dilutions were considered in order to obtain an estimated inhibition ($CFU{\cdot}mL^{-1}$); and by

ii) *fluorescein diacetate staining (FDA)*, non-fluorescent compound hydrolyzed by esterases in fully functional cells to a green fluorescent compound (fluorescein), which is an extensively used indicator for *S. aureus* and *E. coli* enzymatic activity determination. Thus, the liquid fraction was analysed in 96-well black microplates by mixing 5 µL of FDA (2 $mg{\cdot}mL^{-1}$ in dimethyl sulfoxide, DMSO) with 195 µL of bacterial suspension in each well. The plate was incubated at 25 ºC for 30 min, with continuous readings every 5 min (λ_{ex} = 485 nm; λ_{em} = 538 nm) using a fluorometer

(Fluoroskan Ascent FL Fluorimeter/Luminometer; Thermo Scientific, Waltham, MA, USA). The possible fluorescence interference of the culture medium and GR-MOF-7 was also checked.[4] Each sample was measured for quadruple, disclosing the outcomes as an inhibition percentage, calculated as the difference in fluorescent intensity of the sample with respect to one of the control blank assays. Moreover, the fluorometer was also used for the determination of the bacterial oxidation «*ROS production*».[5,6] Briefly, 150 µL of each sample fraction was incubated for 30 min in 96-well black microplates with 50 µL of 10 mol·L^{-1} of the ROS salt (2′,7′-dichlorodihydrofluorescein diacetate, H_2DCF-DA), which is sensitive for hydrogen peroxide and other oxidative species, including hydroxyl and peroxyl radicals. Each sample was measured for quadruple readings, every 5 min (λ_{ex} = 495 nm; λ_{em} = 525 nm) and represented normalized with respect to the negative control group for a direct comparison.

Confocal laser scanning microscopy (CLSM) was performed for visual and qualitative assessment of antibacterial activities. Cell images of each bacteria strain were obtained after the contact with the GR-MOF-7 suspensions (50 µL aliquot) via confocal microscopy using a Confocal SP5 (Leica Microsystems, Germany). The bacteria were stained with a LIVE/DEAD kit (Live/Dead BacLight Viability Kit, Thermo Fisher, USA), which consists in a fluorescent dye, prepared with a mixture of Propodium iodide (PI) and Syto 9 in DMSO (10 µL of each dye and 980 µL of DMSO). This staining permeates the cell membrane depending on its integrity: viable cells exhibit green fluorescence (Syto 9: live cells, λ_{ex} = 480 nm; λ_{em} = 500 nm), whereas non-viable bacteria provide red intensity (PI: dead cells, λ_{ex} = 490 nm; λ_{em} = 635 nm). The fluorescent dye mixture (10 µL) incubation in contact with the different bacteria took place during 30 min in dark at RT.

Bioactivity on pests

Effect on seeds germination

Seeds of *Rabanus satibus* (radish) were purchased from authorized dealer with certification ES-ECO-001-AN European Agriculture Ecojaral – Productos Ecológicos, Granada, Spain (www.ecojaral.com). The average germination rates of plant seed were greater than 90%. Seeds were kept in a dry place in the dark under room temperature before use.

Previous to any test, the active concentration of glufosinate ammonium against *R. sativus,* used in this study as model invasive weed, was determined. The concentration was selected according to the recommendations of the commercial glufosinate based pesticide BASF-Rely280®.[7] Aqueous solutions (5 mL) of glufosinate ammonium with different concentrations (*R. sativus*: $6.35\cdot10^{-3}$, 0.012 and 0.025 M) were tested. In parallel, a water control was also performed. 7 cm^2 Petri dishes were used for each different concentration with a total of 20 seeds *per* petri dish. Radish seed germination was studied for 12 days. During this time, control seeds develop different growth stages (1, 2, 3 and 4), as shown in **Figure S1**.

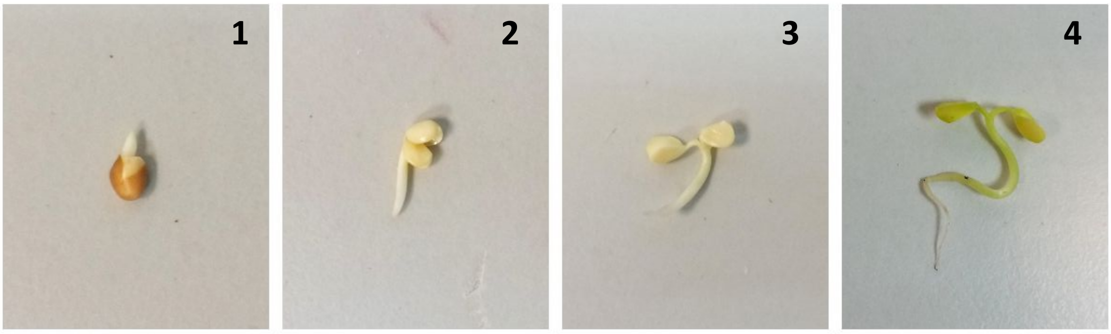

Figure S1. Radish seeds growth stages (1, 2, 3, and 4) when treated with water (negative control) after 7 days.

The number of germinated seeds was counted to obtain the germination rate (GR).

$$GR = \text{seeds germinated/total seeds} \times 100$$

Once the active concentration of glufosinate ammonium was determined, the activity of GR-MOF-7 was studied against *R. sativus*. 7 mL of an aqueous solution of GR-MOF-7 (2.55 $g \cdot L^{-1}$ or 0.010 M) were added to a petri dishes containing 20 weeds following the same procedure as previously described. In parallel, three different controls (water, glufosinate, and glufosinate + $Cu(NO_3)_2 \cdot 3H_2O$) were performed using the same concentration (0.010 M). All tests were carried out at least in triplicate.

Effect on pest (seed and plant) growth

Experiments were conducted in Granada (Spain) under ambient conditions, with an average day/night photoperiod of 15/10 h, and temperature 30/15 ºC and in a dry environment. *R. sativus* germinated seeds were sown in 15 x 10 cm flowerpots containing artificial mixed soil (Compo Sana), and each pot contained 10 plants. *R. sativus* plants were watered daily to optimize their growth. When the plants reach the 3-leaf stage, they were sprayed once with 1 mL of GR-MOF-7 and glufosinate ammonium solution (0.010 M) using a microaerosol sprayer. Water was used as control. All tests were carried out at least in triplicate.

Effect on non-target plant

Experiments were conducted in Granada (Spain) under ambient conditions, with an average day/night photoperiod of 15/10 h, and temperature 40/15 ºC and in a dry environment. *Ribes nigrum* (berry) plants were sprayed once with 1 mL of GR-MOF-7 solution (0.010 M) using a microaerosol sprayer.

Statistics

The results of the different assays are represented as mean ± standard deviation (SD). Ordinary Two-way ANOVA analysis of variance followed by a Tukey´s multiple comparison tests were carried out to determine significant differences using GraphPad Prism 9.2 software (GraphPad Software, Inc., La Jolla, CA, USA). Each experiment was performed at least three times ($n \geq 3$). In the graphs, the results are indicated as: $P > 0.05$, $^{*}P \leq 0.05$, $^{**}P \leq 0.01$, $^{***}P \leq 0.001$ and $^{****}P \leq 0.0001$.

S2. Crystallographic data

Table S1. Crystallographic data and structure refinement details of **GR-MOF-7**.

**Compound**	**GR-MOF-7**
Formula	$C_5H_{10}NO_4PCu$
M_r	242.65
CCDC	2109530
Crystal system	Monoclinic
Space group (no.)	$P2_1/c$
T (K)	120(2)
a (Å)	9.985(3)
b (Å)	4.9674(15)
c (Å)	16.103(4)
α (°)	90.000
β (°)	106.876(10)
γ (°)	90.000
V (Å^3)	764.3(4)
Z	4
ρ_{calc} (g·cm^{-3})	2.109
μ (mm^{-1})	3.037
F(000)	492
Index ranges	$-11 \leq h \leq 11$, $-4 \leq k \leq 5$, $-17 \leq l \leq 17$
Independent reflections	3495 [R_{int} = 0.0548]
Goodness of fit	1.093
Final R indexes [I > 2σ (I)]	R_1 = 0.0325, wR_2 = 0.0699
Final R indexes [all data]	R_1 = 0.0492, wR_2 = 0.0749
Largest diff. peak/hole (e·Å^{-3})	-0.435/0.402

Table S2. Selected bond lengths (Å) and angles (°) for **GR-MOF-7**.

**Bond Distances (Å)**	**Bond Angles (°)**
Cu1-N1 1.977(4)	N1-Cu1-O2 100.65(13)
Cu1-O1 1.952(3)	N1-Cu1-O3 86.97(14)
Cu1-O2 2.274(3)	O1-Cu1-N1 83.16(14)
Cu1-O3 1.979(3)	O1-Cu1-O2 97.42(12)
Cu1-O4 1.935(3)	O1-Cu1-O3 167.47(13)
	O3-Cu1-O2 91.96(12)
	O4-Cu1-N1 165.15(14)
	O4-Cu1-O1 90.68(14)
	O4-Cu1-O2 93.52(12)
	O4-Cu1-O3 97.04(13)

Table S3. Hydrogen bond distances (Å) and angles (°) for **GR-MOF-7**.

D-H···A	Distance (D-H)	Distance (H···A)	Distance (D-H···A)	Angle
N1-H1A···O1	0.95	1.98	2.933(5)	176.6
N1-H1B···O3	0.88	2.09	2.940(5)	161.1

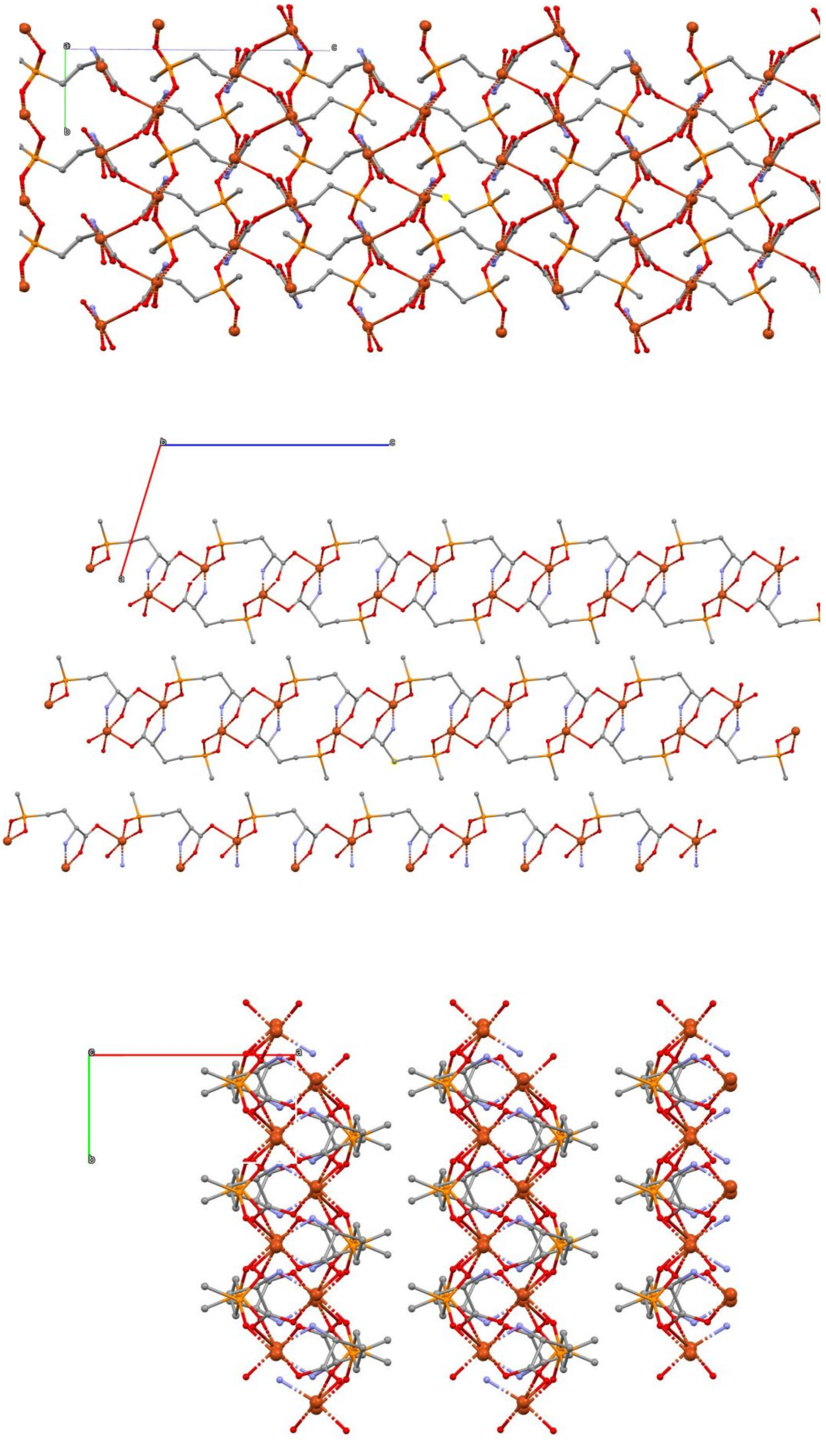

Figure S2. Different perspectives of GR-MOF-7 along *a* (top), *b* (middle) and *c* (bottom) axis.

S3. GR-MOF-7 characterization

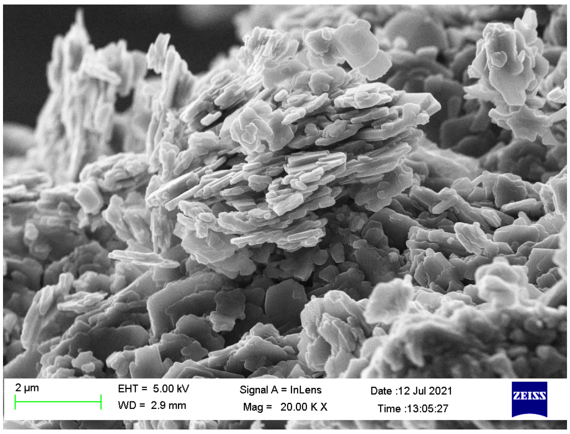

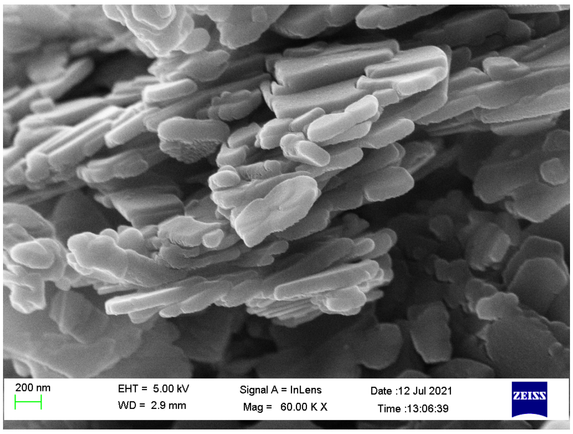

Figure S3. SEM images of GR-MOF-7 tetragonal layered particles of about ~1 µm.

Powder XRD refinement of GR-MOF-7

The lattice parameters were refined using TOPAS software (version 5, Bruker AXS, Karlsruhe, Germany). There is a good agreement between the data and the model.

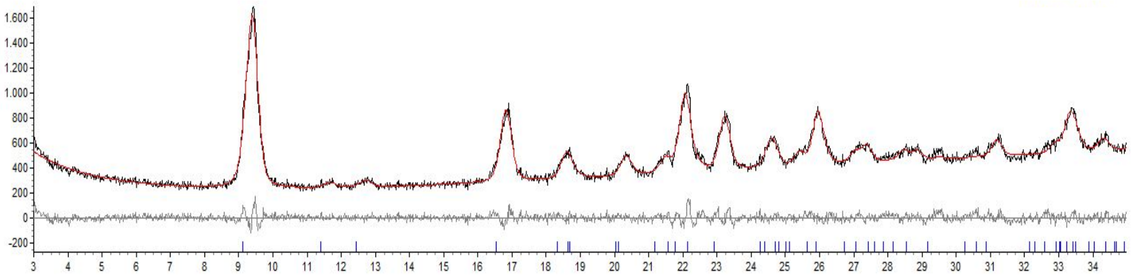

Figure S4. Le Bail fitting of GR-MOF-7.

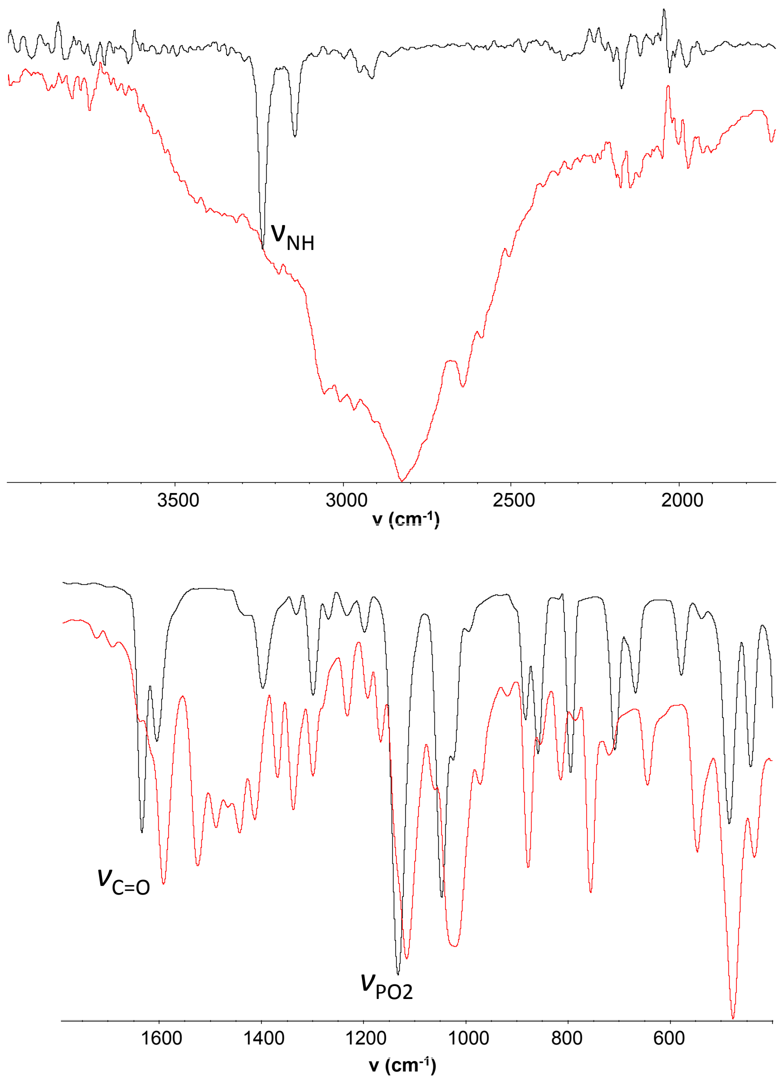

Figures S5. FTIR spectra of the GR-MOF-7 (black) and the glufosinate linker (red).

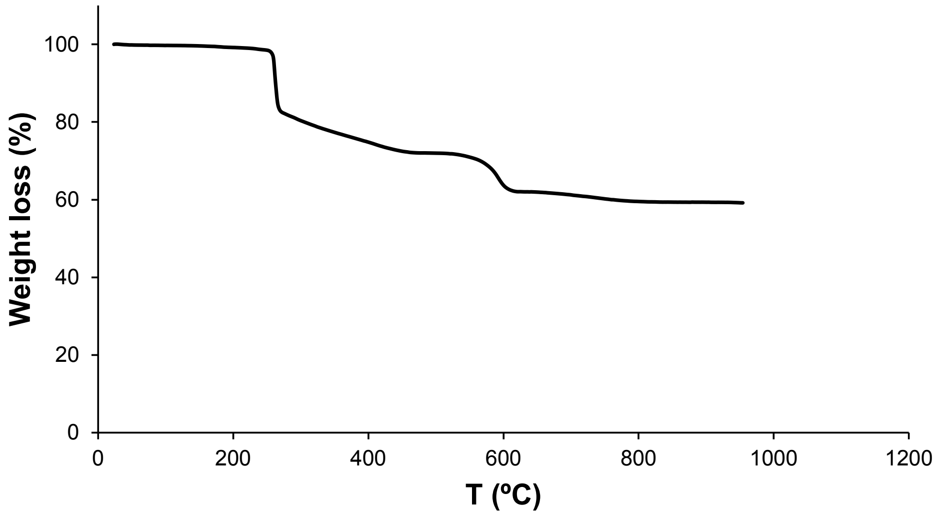

Figure S6. Thermogravimetric analysis (TGA) of the GR-MOF-7 material.

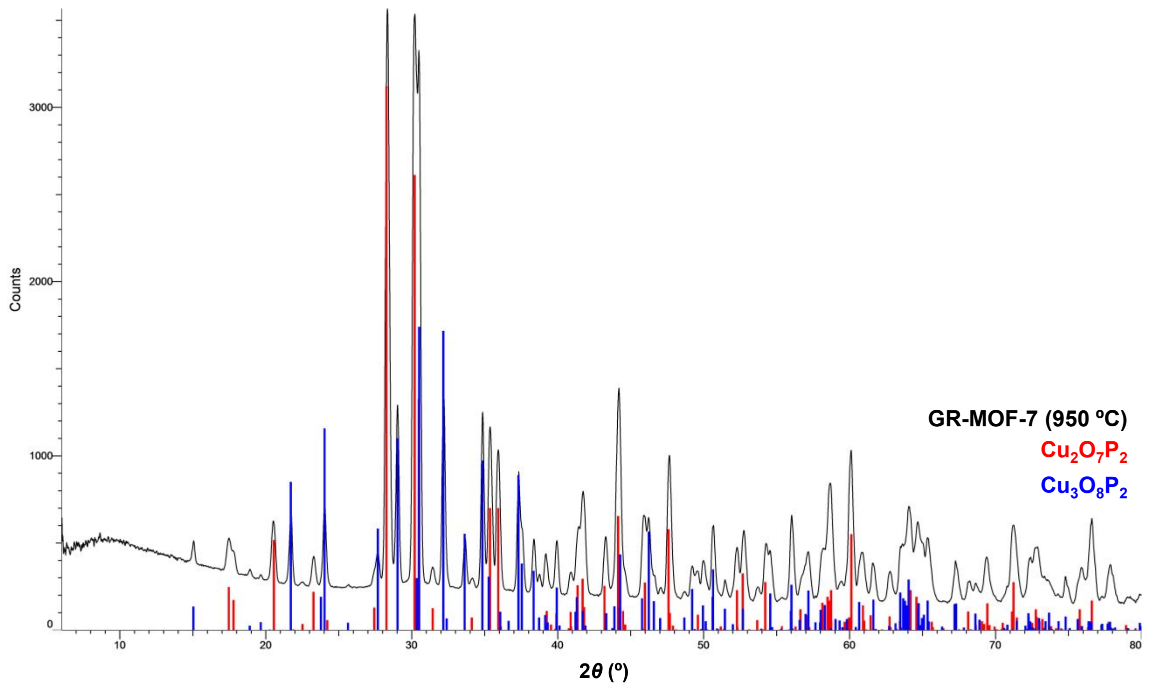

Figure S7. Powder X-ray diffraction (XRD) patterns of GR-MOF-7 (black) after been heated up to 950 ºC under air, and its identified residual species $Cu_3O_8P_2$ (blue) and $Cu_2O_7P_2$ (red).

S4. Aqueous stability studies of GR-MOF-7

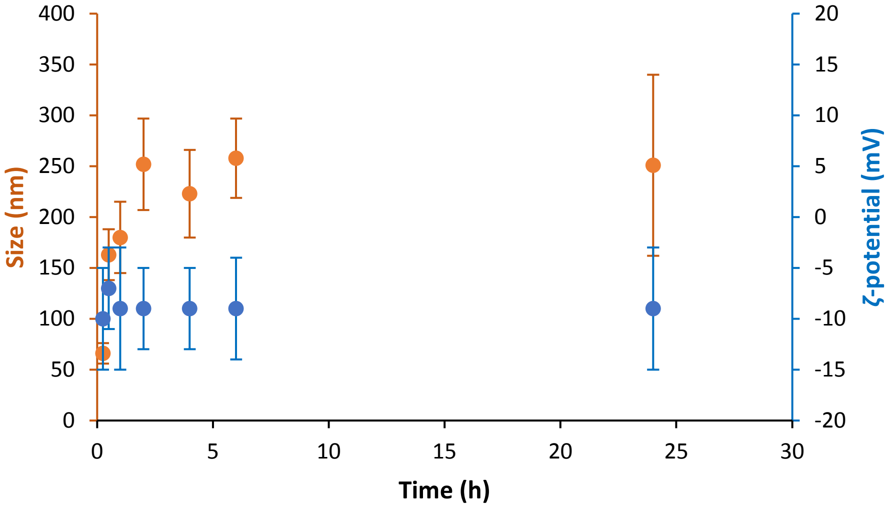

Figure S8. Colloidal stability of GR-MOF-7 in aqueous solution (24h, RT). Stability is represented as the average of particle size (left, orange) and ζ-potential (right, blue) evolution over 24 h.

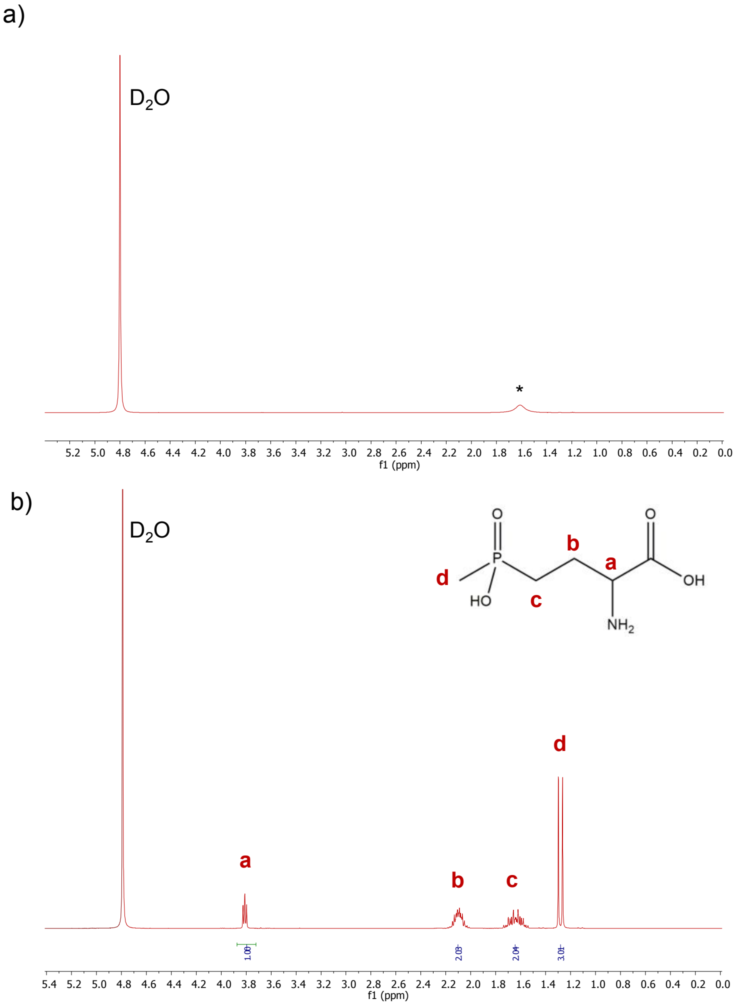

Figure S9. ^{1}H NMR of solutions of (a) the paramagnetic GR-MOF-7 and (b) glufosinate in D_2O. $^{*}CDCl_3$ impurity

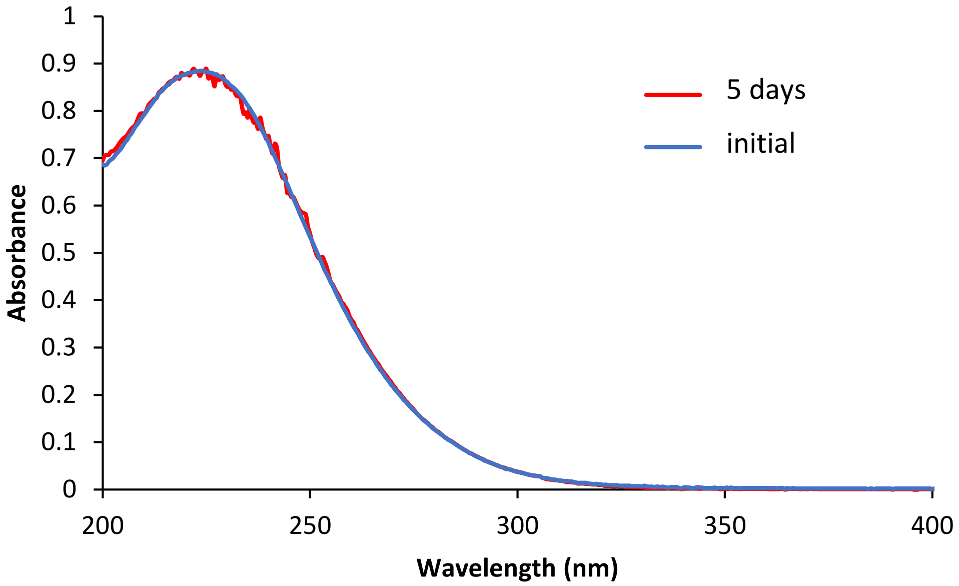

Figure S10. Comparison between the UV-vis spectra of a fresh prepared water solution of GR-MOF-7 and after 5 days aqueous suspension.

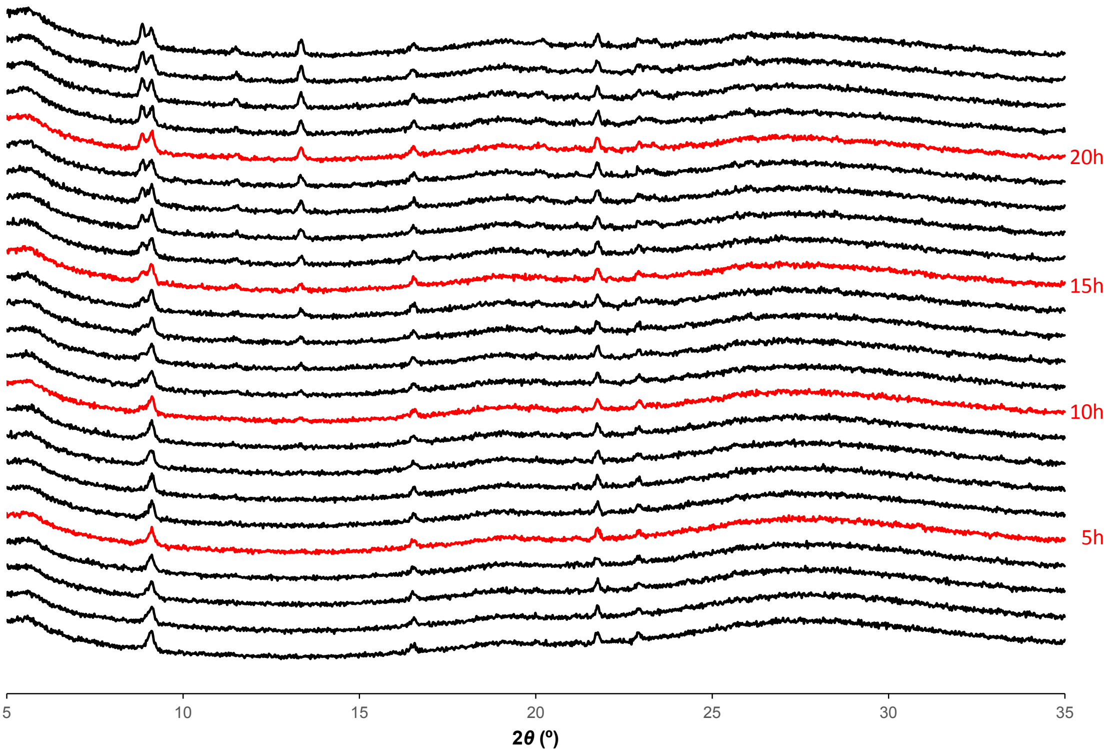

Figure S11. Powder X-ray diffraction (XRD) patterns of GR-MOF-7 under wet conditions for 24 h.

S5. Antibacterial effect

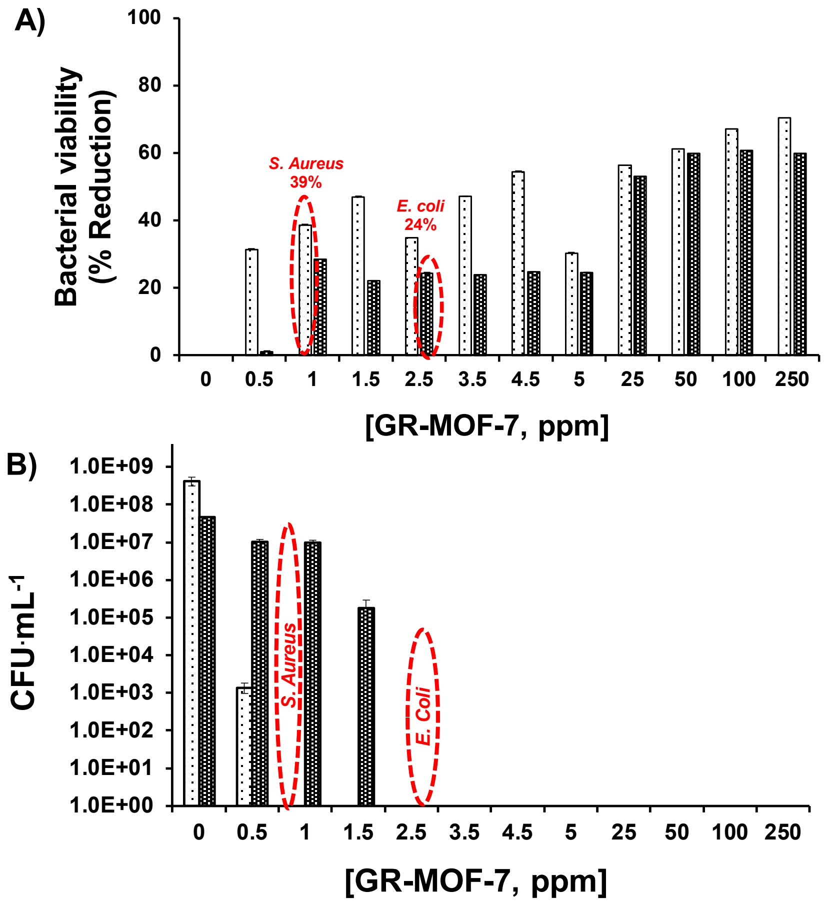

Figure S12. *S. aureus* (white column) and *E. coli* (black column) viability after 20 h of contact with a wide range of GR-MOF-7 concentrations determined by fluorescein diacetate hydrolysis assay (FDA; A) and colony-forming unis (CFU; B). In all cases, each sample value was normalized with a negative control (C-, 100% of bacterial viability).

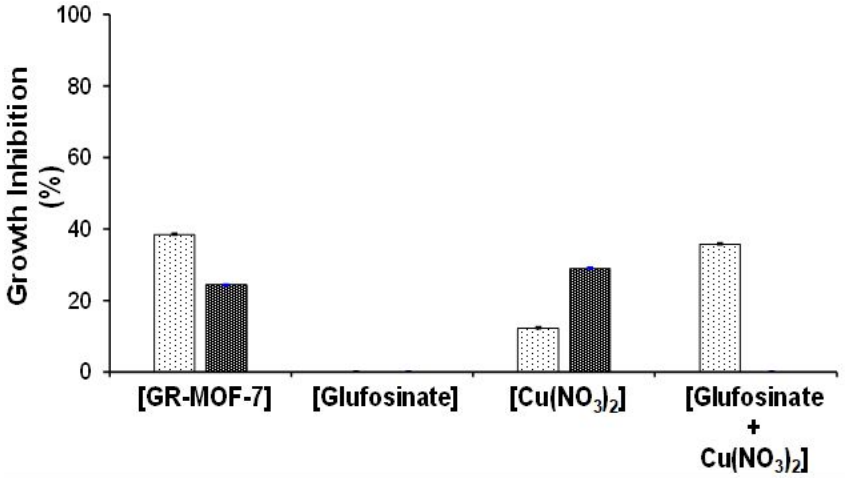

Figure S13. Enzymatic activity loss (%) of *S. aureus* (white column) and *E. coli* (black column) after 20 h of contact with the selected active GR-MOF-7 concentration together with the corresponding amount of the following controls: $Cu(NO_3)_2$, free glufosinate and a precursor mixture. In all cases, each sample value was normalized with a negative control (C-, 100% of bacterial viability). No statistical significant differences were observed.

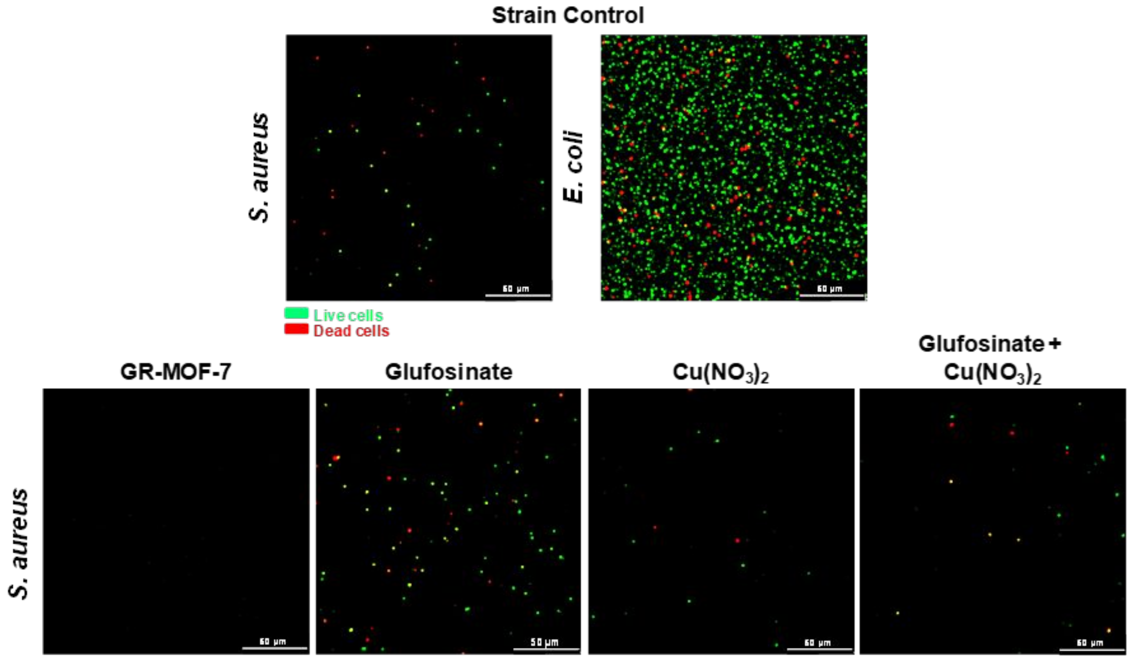

Figure S14. Fluorescence LIVE/DEAD confocal images of sessile *S. aureus* on cover glasses surface after 20 h of contact with GR-MOF-7 compound and its constituents, used as controls: $Cu(NO_3)_2$, free glufosinate and a precursor mixture. Moreover, both *S. aureus* and *E. coli* controls were also depicted for better comparison. The scale bar corresponds to 50 µm. All the images were taken at 63X.

S7. Herbicidal effect

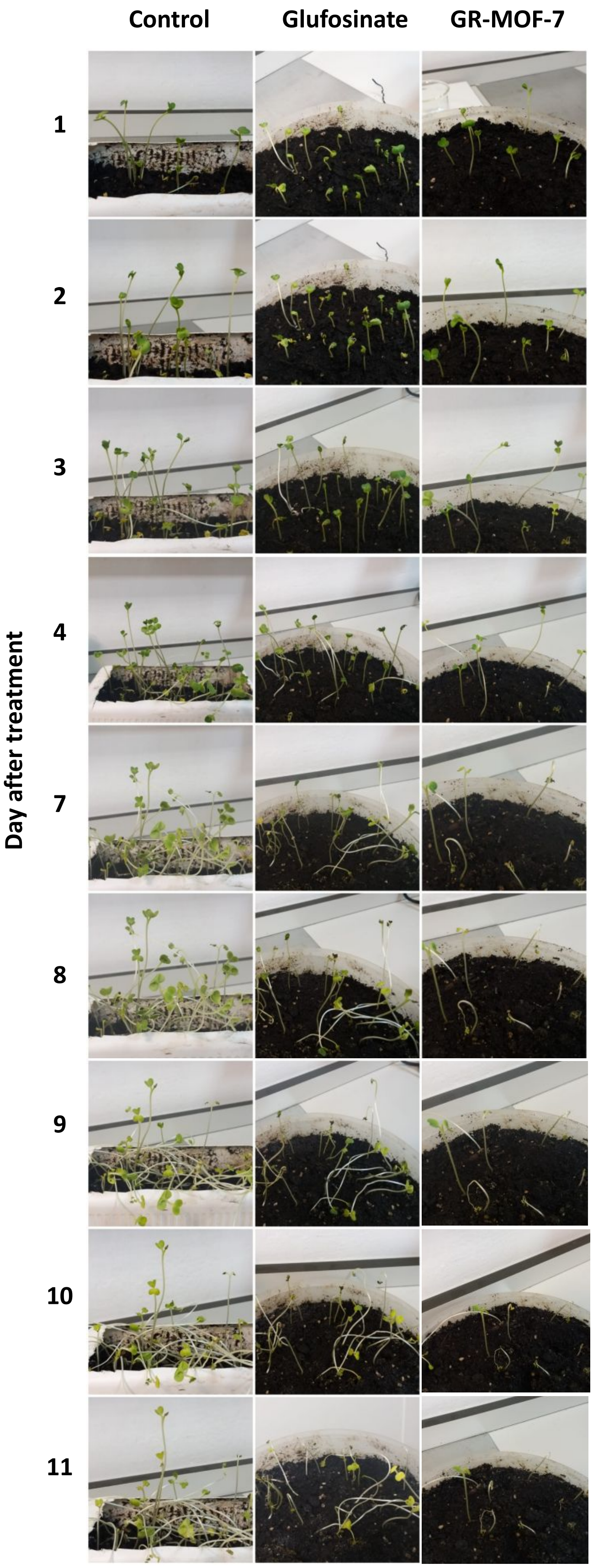

Figure S15. Effect of free glufosinate and GR-MOF-7 on grown radish plants for 11 days.

GR-MOF-7

1

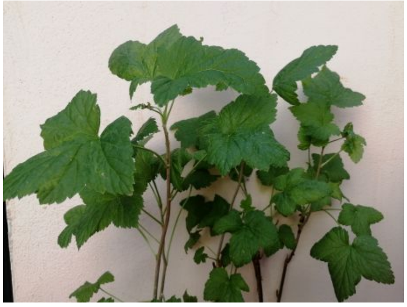

7

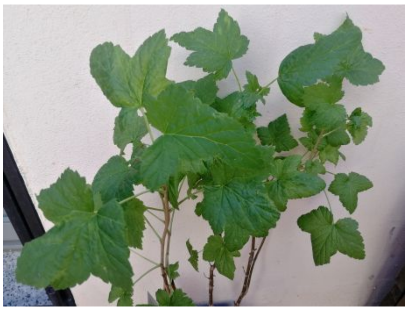

11

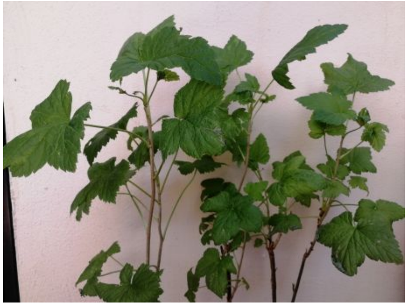

Figure S16. Effect of GR-MOF-7 on *Ribes nigrum* for 11 days.

S6. References

(1) Bruker AXS Inc.: Madison. Burker Apex2. B. A. I. Bruker Apex2. WI, USA 2004.

(2) Sheldrick, G. M. Program for Empirical Adsorption Correction. 1996.

(3) Sheldrick, G. M. SHELXT - Integrated Space-Group and Crystal-Structure Determination. *Acta Crystallogr. Sect. A Found. Crystallogr.* **2015**, *71* (1), 3–8.

(4) Clarke, J. M.; Gillings, M. R.; Altavilla, N.; Beattie, A. J. Potential Problems with Fluorescein Diacetate Assays of Cell Viability When Testing Natural Products for Antimicrobial Activity. *J. Microbiol. Methods* **2001**, *46* (3), 261–267.

(5) Amariei, G.; Kokol, V.; Vivod, V.; Boltes, K.; Letón, P.; Rosal, R. Biocompatible Antimicrobial Electrospun Nanofibers Functionalized with ε-Poly-L-Lysine. *Int. J. Pharm.* **2018**, *553* (1–2), 141–148.

(6) Arenas-Vivo, A.; Amariei, G.; Aguado, S.; Rosal, R.; Horcajada, P. An Ag-Loaded Photoactive Nano-Metal Organic Framework as a Promising Biofilm Treatment. *Acta Biomater.* **2019**, *97*, 490–500.

(7) Out, K.; Reach, O. F.; Children, O. F. Rely280. 2016.
